# Two-stage algorithms for visually exploring spatio-temporal clustering of avian influenza virus outbreaks in poultry farms

**DOI:** 10.1038/s41598-021-01207-4

**Published:** 2021-11-19

**Authors:** Hong-Dar Isaac Wu, Day-Yu Chao

**Affiliations:** 1 Department of Applied Mathematics and Institute of Statistics, National Chung Hsing University, Taichung, Taiwan; 2 Graduate Institute of Microbiology and Public Health, College of Veterinary Medicine, National Chung Hsing University, Taichung, 402 Taiwan

**Keywords:** Infectious diseases, Influenza virus

## Abstract

The development of visual tools for the timely identification of spatio-temporal clusters will assist in implementing control measures to prevent further damage. From January 2015 to June 2020, a total number of 1463 avian influenza outbreak farms were detected in Taiwan and further confirmed to be affected by highly pathogenic avian influenza subtype H5Nx. In this study, we adopted two common concepts of spatio-temporal clustering methods, the Knox test and scan statistics, with visual tools to explore the dynamic changes of clustering patterns. Since most (68.6%) of the outbreak farms were detected in 2015, only the data from 2015 was used in this study. The first two-stage algorithm performs the Knox test, which established a threshold of 7 days and identified 11 major clusters in the six counties of southwestern Taiwan, followed by the standard deviational ellipse (SDE) method implemented on each cluster to reveal the transmission direction. The second algorithm applies scan likelihood ratio statistics followed by AGC index to visualize the dynamic changes of the local aggregation pattern of disease clusters at the regional level. Compared to the one-stage aggregation approach, Knox-based and AGC mapping were more sensitive in small-scale spatio-temporal clustering.

## Introduction

In Asia, Europe, and North America, the emergence and intercontinental spread of the highly pathogenic avian influenza A (HPAI) H5Nx virus clade 2.3.4.4 has caused substantial economic losses to the poultry industry^1^. As a critical stop-over site for migratory birds along the flyway of Asia, Taiwan has experienced epidemics caused by multiple introductions of different HPAI novel subtypes of clade 2.3.4.4 virus in the past years^2,3^, making the control of the spread of avian influenza a challenging issue in public health. Due to the extreme vulnerability of the poultry industry and its potential economic losses, a sensitive statistical tool for the timely identification of clusters and visualization of their dynamic change would be invaluable in implementing more stringent control measures and determine potential factors, such as vehicle transportation or wild birds, for virus spread.

Essential steps in identifying spatial clusters of any infectious disease, such as HPAI, include (1) identification of areas having exceptionally high (or low) events; (2) determination of whether the abnormal counts of events can be attributed to chance variation or is statistically significant; (3) assessment of explanatory factors that may account for such abnormal clustering. However, accurately identifying the spatial clusters and predicting the direction of viral spread requires the knowledge of several environmental factors with spatial structures, which are not only non-randomly distributed across a country but are also changing with time. There are many research articles devoted to modeling the spatial and temporal diffusion of various infectious diseases^4–8^. Although some of them applied complex spatial statistics to detect a series of epidemiological anomalies, the most popular method is spatial scan statistics proposed by Kulldorff in 1997^9^. The SaTScan software, developed by Kulldorff et al. in 1998, applying space–time scan statistics with the option of different distributions, such as binomial or Poisson, has been widely used globally. However, the disadvantages of scan statistics are (1) the need to have the prior knowledge of a reference population; (2) the arbitrary selection of a window size for reference population and time, which requires trial-and-error to find the optimal cutoff value; (3) the events within the pre-defined space or time prevent application to the detection of abnormal events under a certain network, such as highway or water supply. On the contrary, another commonly used method, the Knox test, can determine the proper scale of space and time objectively without the prior knowledge of a reference population^10,11^.

In this study, we improved two common concepts of spatiotemporal clustering methods, including the Knox test and scan statistics, and proposed the two-stage algorithms for visually exploring spatio-temporal clustering, using HPAI poultry farm outbreaks during 2015 in Taiwan as the example. The first two-stage algorithm performs the Knox test followed by the standard deviational ellipse (SDE) method^12–15^ and the second algorithm applies scan likelihood ratio statistics followed by AGC index to visualize the dynamic changes of the local aggregation pattern of disease clusters at the regional level. Both SDE and AGC maps along a regular time interval provide the visual ways of indicating the direction of virus transmission.

## Materials and methods

### Dataset

The details of the dataset collections including outbreak poultry farms and total poultry distribution were described previously^2^. In short, the data of HPAI outbreaks used in this study were collected based on both the nationwide mandatory clinical disease reporting system (CDRS) and active surveillance program, implemented by the Bureau of Animal and Plant Health Inspection and Quarantine (BAPHIQ), Council of Agriculture (COA). The nationwide CDRS was established in 2003 due to the first H5N2 poultry farm outbreaks in Taiwan. All poultry farmers are mandated to report poultry health problems or unusual increases in mortality events to local animal disease control centers (LDCCs), which are further compiled by BAPHIQ. The total poultry farm census data was obtained by spatially merging the official poultry farm registration database (OPFRD) managed by the COA, with an island-wide domestic waterfowl farms survey conducted by the Taiwan Agriculture Research Institute (TARI) utilizing remote satellite imaging technology between August 2016 and April 2017. All data were projected in TWD97/TM2 zone 121 and geocoded using WGS84 datum by ArcGIS, version 10.3 (ESRI, Redlands, CA, USA) for mapping and visualization with high resolution. From 2015 to June 2020, the total number of avian influenza outbreak farms with laboratory-confirmation is 1,463. Since most (68.6%) of the outbreak farms were detected in 2015, only the data in 2015 was used for analysis in this study.

### Knox method

Based on the method proposed by Knox^10^, we searched all possible pairs close in space and time to obtain the maximum odds ratio to generate the optimum association. To assess the significance, no rigorous statistical method has been developed as a formal test to give an exact or approximate (in terms of large sample theory) p-value for two-dimensional searching, i.e. space–time in this case, for optimal cutoff points, although Mantel (1967) proposed to use the permutation methods^16^. However, a formal test is not what we pursued here. Instead, a visual technique to explore spatio-temporal clustering of a reasonably exhibited pattern has been developed as stated below.

The construction of Knox statistics involves the partitions of time and space. Let **A** be the area under study, and **T** = [0,τ] with τ being the maximal observation time. Here we explain how to find the “optimal” cut points for distance measured in **A** and for time interval **T**. Consider a series of events that occurred within **A** $\times$ **T**, and let Ω(w,t) be the collection of these events (occurred at 0 < t_1_ < t_2_ < ⋯ < t_n_<τ):

$$\Omega \left( {{\text{w}},{\text{t}}} \right) = \{ {\text{w}}_{{\text{i}}} \left( {{\text{t}}_{{\text{i}}} ;{\text{x}}_{{\text{i}}} ,{\text{y}}_{{\text{i}}} } \right):{\text{t}}_{{\text{i}}} \in {\mathbf{T}}, \, \left( {{\text{x}}_{{\text{i}}} ,{\text{y}}_{{\text{i}}} } \right) \in {\mathbf{A}},{\text{ i}} = {1},{2}, \ldots ,{\text{n}}\} ,$$

where (x_i_,y_i_) is the location of the point w_i_; usually x_i_ is the longitude and y_i_ the latitude. Consider “all pairs” of events (w_i_,w_j_), i ≠ j; there were N = n(n − 1)/2 pairs. Let t_0_ $\in$ **T** and d_0_ be possible cutoff points of time and distance measured for all $\left({w}_{i},{w}_{j}\right)$-pairs. Suppose m, s, and a_0_ satisfy:

$$\sum_{\text{i}>j}\sum_{\text{j}=1}^{\text{n}}{1}_{\{{\text{t}}_{\text{i}}-{\text{t}}_{\text{j}}<{\text{t}}_{0}\}}={\text{m}}_{0}, \sum_{\text{i}>j}\sum_{\text{j}=1}^{\text{n}}{1}_{\{\text{d}\left({\text{w}}_{\text{i}},{\text{w}}_{\text{j}}\right)<{\text{d}}_{0}\}}={\text{s}}_{0} ,\text{ and}$$

$$\sum_{\text{i}>j}\sum_{\text{j}=1}^{\text{n}}{1}_{\{{\text{t}}_{\text{i}}-{\text{t}}_{\text{j}}<{\text{t}}_{0},\text{d}\left({\text{w}}_{\text{i}},{\text{w}}_{\text{j}}\right)<{\text{d}}_{0}\}}={\text{a}}_{0},$$

where ${1}_{A}$ is an indicator variable for event “A”. An odds ratio is calculated by OR_0_ = a_0_d_0_/b_0_c_0_ from the following 2 $\times$ 2 table:

**Table:** 

Number of pairs	$d\left({w}_{i},{w}_{j}\right)<{d}_{0}$	$d\left({w}_{i},{w}_{j}\right)>{d}_{0}$	
t_i_ − t_j_ < t_0_	${a}_{0}$	${b}_{0}$	${m}_{0}$
t_i_ − t_j_ > t_0_	${c}_{0}$	${d}_{0}$	N-${m}_{0}$
	${s}_{0}$	N-${s}_{0}$	N

With the cutoff (t_0_,d_0_), the table above shows that there are ${a}_{0}$ pairs judged as being close in space and time. The choice of t_0_ and d_0_ is not really arbitrary, we will search all possible values of t_0_ and d_0_ to obtain the maximum odds ratio to generate the maximum association. We express it as OR_max_ = a_1_d_1_/b_1_c_1_, where (a_1_,b_1_,c_1_,d_1_) is the number of pairs of the corresponding cell. Therefore, the optimal cutoff value of space and time is determined based on OR_max._

To explore spatio-temporal clustering, the a_1_ pairs of events considered to be close to each other (in time and space) were connected with line segments, one segment for on pair. Once the optimality was determined, the independent clusters could be decided visually. Here “independence” is not used in the strict definition of probability, rather, is determined arbitrarily or visually as described in the Supplementary Information. Once the *major clusters* were determined, the other smaller clusters were treated as *minor clusters*. The major or minor clusters were defined based on the size of the clusters, to be specific, the numbers of outbreak farms within the clusters. In this study, the major clusters contained at least 10 outbreak poultry farms, and clusters that contained between 5 and 9 outbreak farms were called minor clusters. The SDE method was implemented to reveal the transmission direction for each spatial cluster.

### Likelihood ratio-based method

Instead of applying space–time scan statistics, the hierarchical clustering method using second-order likelihood-based scan statistics was performed in this study. Suppose that the surveyed region, denoted as **g,** is a sub-region of “population area” **G**. For example, **G** can be the entire country, and **g** can be a specific administrative unit (such as a county). Further, {A_j_} is a set of townships that forms a partition of **g**:

$$\mathbf{g}={A}_{1}\cup {A}_{2}\cup \dots \cup {A}_{K}.$$

**Figure 1:**
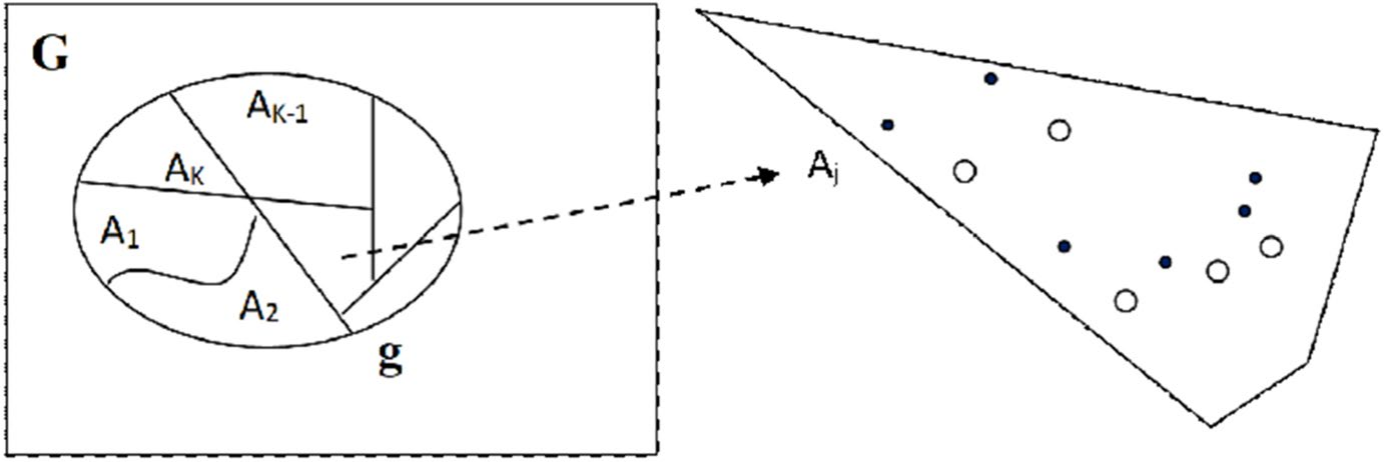
A scheme for investigated region **g** with partition {A_j_} and reference population **G**. In some A_j_, for example, a black dot denotes the “spot” of an outbreak farm while a circle represents a non-outbreak. Within a defined period, there were a total of T_j_ poultry farms with N_j_ outbreaks. Notice that if A_j_ is the focus and assuming heterogeneity, **g** can also be the reference.

Let the total number of poultry farms in the sub-region A_j_ be T_j_, and there are N_j_ outbreaks in the defined time interval within a township A_j_. Further, N_G_ is the number of outbreaks in G, and T_G_ is the corresponding total number. Figure 1 gives a scheme for the relationship between the investigated region **g**, with partition {A_j_}, and the general population **G**. Here, we treat the outbreak probabilities {${P}_{{A}_{j}}$}(j = 1,…,k) as a set of heterogeneous incidence probabilities. Due to substantial heterogeneity in outbreak probabilities, the K null hypotheses ${H}_{0j}^{\left(G\right)}:{P}_{{A}_{j}}={P}_{G}$ versus ${H}_{a}^{(G)}: {P}_{{A}_{j}}>{P}_{G}$ were considered separately, and the log-likelihood ratio

1 $${\lambda }_{j,G}=2\{log{L}_{j}\left({P}_{{A}_{j}},{P}_{G}\right)-log{L}_{0}\left({P}_{C}\right)\}$$

were obtained by a manner similar to those given in Kulldorff (1997) and Duczmal et al. (2006)^9,17^ with ${\widehat{P}}_{G}=\frac{{(N}_{G}-{N}_{j})}{\left({T}_{G}-{T}_{j}\right)},$ ${\widehat{P}}_{{A}_{j}}=\frac{{N}_{j}}{{T}_{j}}$ and ${\widehat{P}}_{C}={N}_{G}/{T}_{G}$ under the likelihoods ${L}_{j}\left(t\right)={P}_{{A}_{j}}^{{N}_{j}}{\left(1-{{P}_{A}}_{j}\right)}^{{T}_{j}-{N}_{j}}{P}_{G}^{{N}_{G}-{N}_{j}}{(1-{P}_{G})}^{{T}_{G}-{{T}_{j}-(N}_{G}-{N}_{j})} \,\text{and}$ ${{L}_{0}\left({P}_{c}\right)={\left({P}_{C}\right)}^{{N}_{G}}(1-{P}_{C})}^{{T}_{G}-{N}_{G}}$.

A question arises how to choose **g** as the “reference.” This consideration leads to the hypotheses parallel with the above hypotheses:

$${H}_{0j}^{(g)}: {P}_{{A}_{j}}={P}_{g},\text{ vs}. {H}_{a}^{(g)}:{P}_{{A}_{j}}>{P}_{g}.$$

The local likelihood calculated for the “j-th” sub-region is relative to the reference region **g** and is no longer relative to **G**. This **g** is usually a much smaller area (compared to **G**) and is suspected to be an administrative unit with a relatively high incidence of avian influenza. Therefore, the recalculated individual likelihood using **g** as the new reference population becomes

$${L}_{j}({P}_{{A}_{j}},{P}_{g})={{P}_{{A}_{j}}}^{{N}_{j}}{{(1-P}_{{A}_{j}})}^{{T}_{j}-{N}_{j}}{P}_{g}^{{N}_{g}-{N}_{j}}{(1-{P}_{g})}^{\left({T}_{g}-{T}_{j}\right)-\left({N}_{g}-{N}_{j}\right),}$$

with ${N}_{g}={\sum }_{1}^{K}{N}_{j}$ and ${T}_{g}={\sum }_{1}^{K}{T}_{j}$; N_g_ is the number of outbreaks and T_g_ is the total number of farms, respectively, within **g**. Furthermore, P_g_ is the probability of incidence estimated, under ${H}_{a}^{(g)}$, by ${\widehat{P}}_{g}={(N}_{g}-{N}_{j})/({T}_{g}-{T}_{j})$. On the other hand, under ${H}_{0j}^{(g)}$, ${P}_{{A}_{j}}={P}_{g}\equiv {P}_{S}.$ The likelihood calculated under ${H}_{0j}^{(g)}$ is ${L}_{0}({P}_{S})={{P}_{S}}^{{N}_{g}}{(1-{P}_{S})}^{{T}_{g}-{N}_{g}}$ with ${\widehat{P}}_{S}={N}_{g}{/T}_{g}$ Consequently, the log-likelihood ratio is

2 $${\lambda }_{j,g}=2\left\{log{L}_{j}\left({P}_{{A}_{j}},{P}_{g}\right)-log{L}_{0}\left({P}_{S}\right)\right\}.$$

When using different reference levels (**G** or **g**) to calculate likelihood ratio statistics, comparing the paired values of (${\lambda }_{j,G}$, ${\lambda }_{j,g}$) on $\cup {\{A}_{j}\}$ can reveal how the sub-regions with high incidence rates contribute to the calculation of spatial clustering. If the incidence rates of adjacent A_i_ and A_j_ ($i\ne j$) are both high, they also have similarly high λ-values. We call this phenomenon “aggregation,” which is known as *second-order clustering*.

However, the likelihood ratio evaluated in (1) or (2) has an inherent property, that is, compared with the “average incidence” under the homogeneity assumption, data with extremely low and extremely high incidences will have similar contributions to the likelihood ratio value (λ). For this reason, a reasonable measure that can distinguish between high and low incidence sub-regions is to compare the naïve difference between ${\lambda }_{j,G}$ and ${\lambda }_{j,g}$:

3 $${R}_{j}={\lambda }_{j,G}-{\lambda }_{j,g}.$$

We call ${R}_{j}$ the AGC index. Generally, unless the statistical hypotheses were ill-posed, the λ-value should be positive since in formula (1), $log{L}_{j}\left({P}_{{A}_{j}},{P}_{G}\right)$ is always larger than $log{L}_{0}\left({P}_{C}\right)$ when the maximum likelihood is used; and similarly, in formula (2), $log{L}_{j}\left({P}_{{A}_{j}},{P}_{g}\right)$ is always larger than $log{L}_{0}\left({P}_{S}\right)$. If the area **g** $=\cup {\{A}_{j}\}$ we calculated or scanned an in-average high incidence area, then ${R}_{j}$ tends to be positive in the sub-region ${A}_{j}$ where the incidence is significantly higher and negative in the sub-region where the incidence is lower. Therefore, the well-known hierarchical clustering method can be used to perform one-dimensional clustering on ${\{{R}_{j}\}}_{j=1}^{K}$ to depict a complete “AGC map”.

## Results

### Spatial–temporal distribution of HPAI outbreak poultry farms

**Figure 2:**
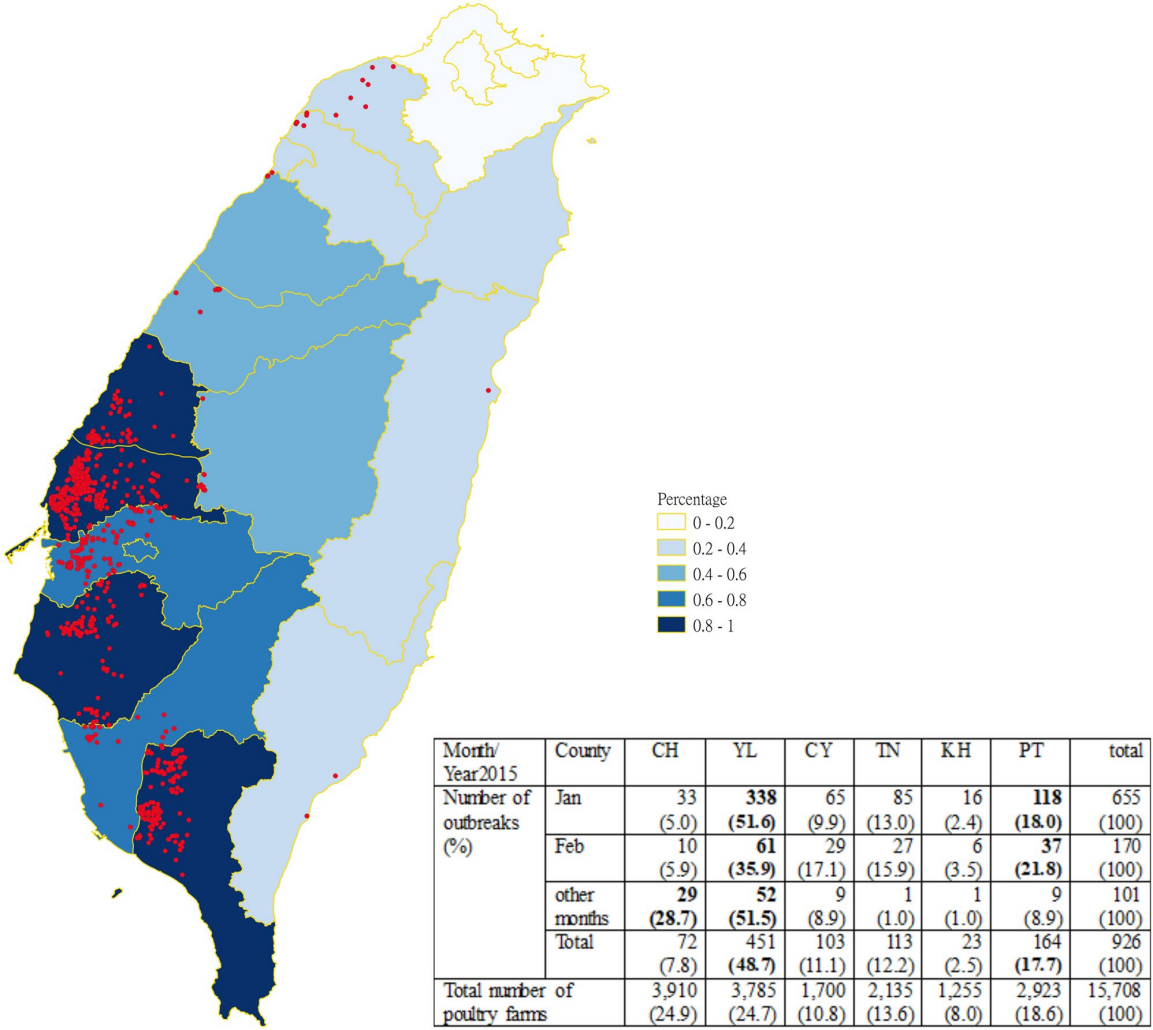
The geographical distribution map of poultry farm outbreaks in Taiwan from 2015. There were 926 outbreaks in total (red dots). Due to the high incidence of the six counties in the southwest, we use a darker frame to mark their locations. The number in the lower right corner of the figure represents the number of poultry farm outbreaks and the total numbers of poultry farms of the six investigated counties in southwestern Taiwan in the year of 2015. The percentages were calculated based on the total outbreaks in each county relative to the total number of poultry farms per month and the year total. County names: *CH* Chang-Hwa, *YL* Yun-Lin, *CY* Chia-Yi, *TN* Tai-Nan, *KH* Kao-Hsiung, *PT* Pin-Tung. Each county was colored based on the percentage calculated by using the number of poultry farms from each county divided by the total poultry farms in Taiwan.

Taiwan, the Asian flyway of migrating birds, experienced the largest poultry epidemic caused by HPAI H5 virus clade 2.3.4.4 in January 2015. A total number of 926 outbreak poultry farms were confirmed by laboratory diagnosis in 2015. The geographical distribution of the outbreak farms is shown in Fig. 2. Overall, Yun-Lin (YL) County had the highest number of outbreaks (48.7%), and Ping-Tong (PT) had the second highest (17.7%). Most outbreaks occurred in southwestern Taiwan, which is a major location for poultry farms with nearly 50% of them situated in YL and Chang-Hwa (CH) counties. The monthly numbers in the six counties with abbreviations were listed in the table within Fig. 2. Partly due to the culling policy, which largely depleted the poultry farm population, 89.1% of the outbreak cases occurred in January and February across the six counties; hence, these two months were selected for temporal and spatial mapping in this study.

### Knox-based spatio-temporal clustering

**Figure 3:**
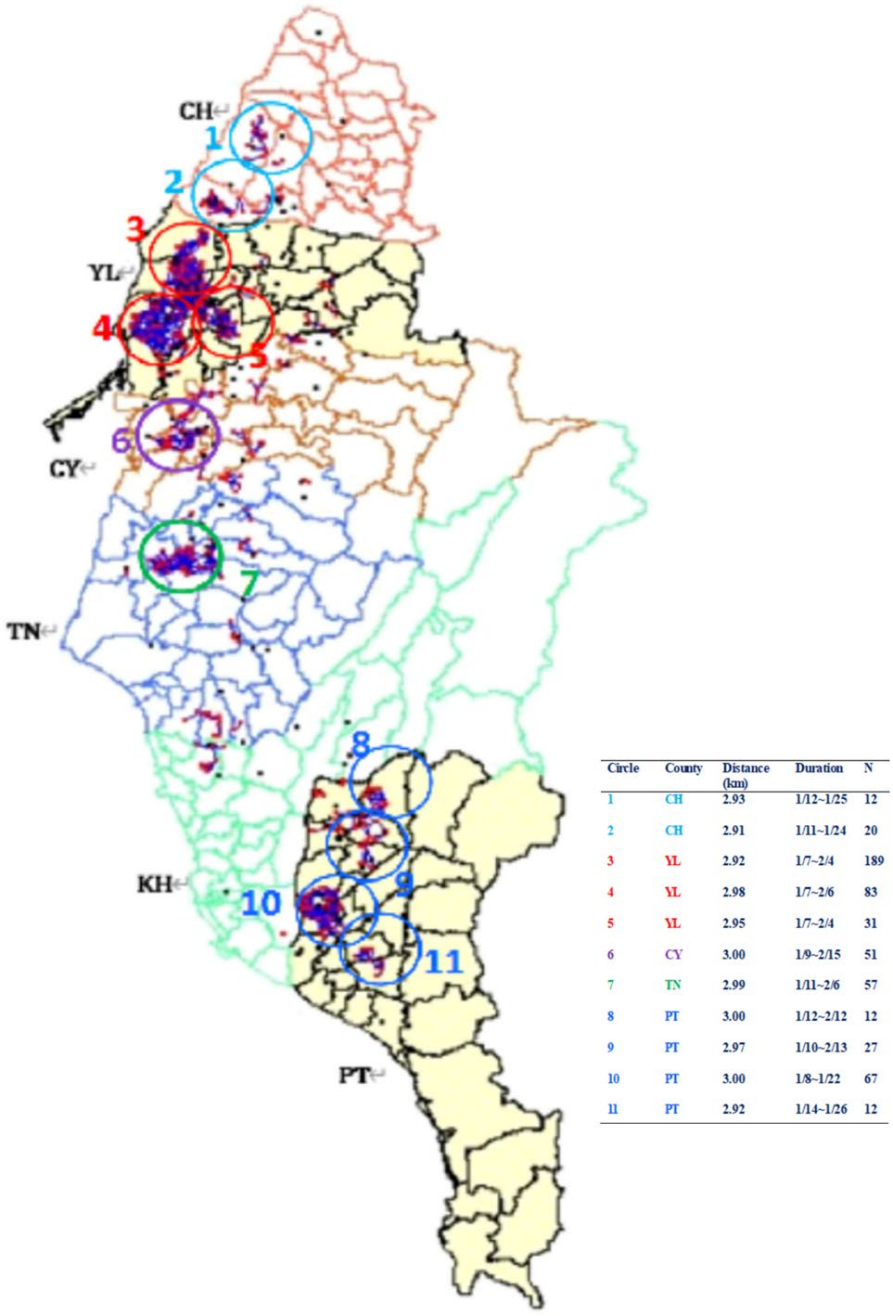
The partition of 6 counties (southwestern Taiwan) formed by Knox statistic under optimal space–time cut-off; 11 major clusters are identified. For each circle, which represents a cluster, the distance (reported in the table) is defined as the maximum distance (kilometer, km) among all of the outbreak pairs. Duration denotes the dates between the first and the last outbreak farms in the circle, and N is the number of outbreaks in that circle.

The implementation of the Knox statistic suggests that the optimal cutoff point for spatial distance is 3 km while that for time is 7 days with details described in Supplementary Information. By connecting pairs with line segments, we have visually identified spatial clustering from the appearance of the “bunches” of the overlapping part of the line segments. “Independent clustering” was not assessed by the strict definition of probability; instead, we define it by drawing the connecting lines, and the lines  between the main clusters is better to be as sparse as possible. In this study, we used the arbitrary numbers of those clusters with “0” or smaller than “5” segments connected to each other to be identified as being “independent”. Therefore, a total of 11 major clusters were determined in the six counties with 2 in CH, 3 in YL, 1 in CY, 1 in TN, 0 in KS, and 3 in PT county. Additionally, there are at least five other minor clusters, but these are not easy to be clearly defined (Fig. 3).

**Figure 4:**
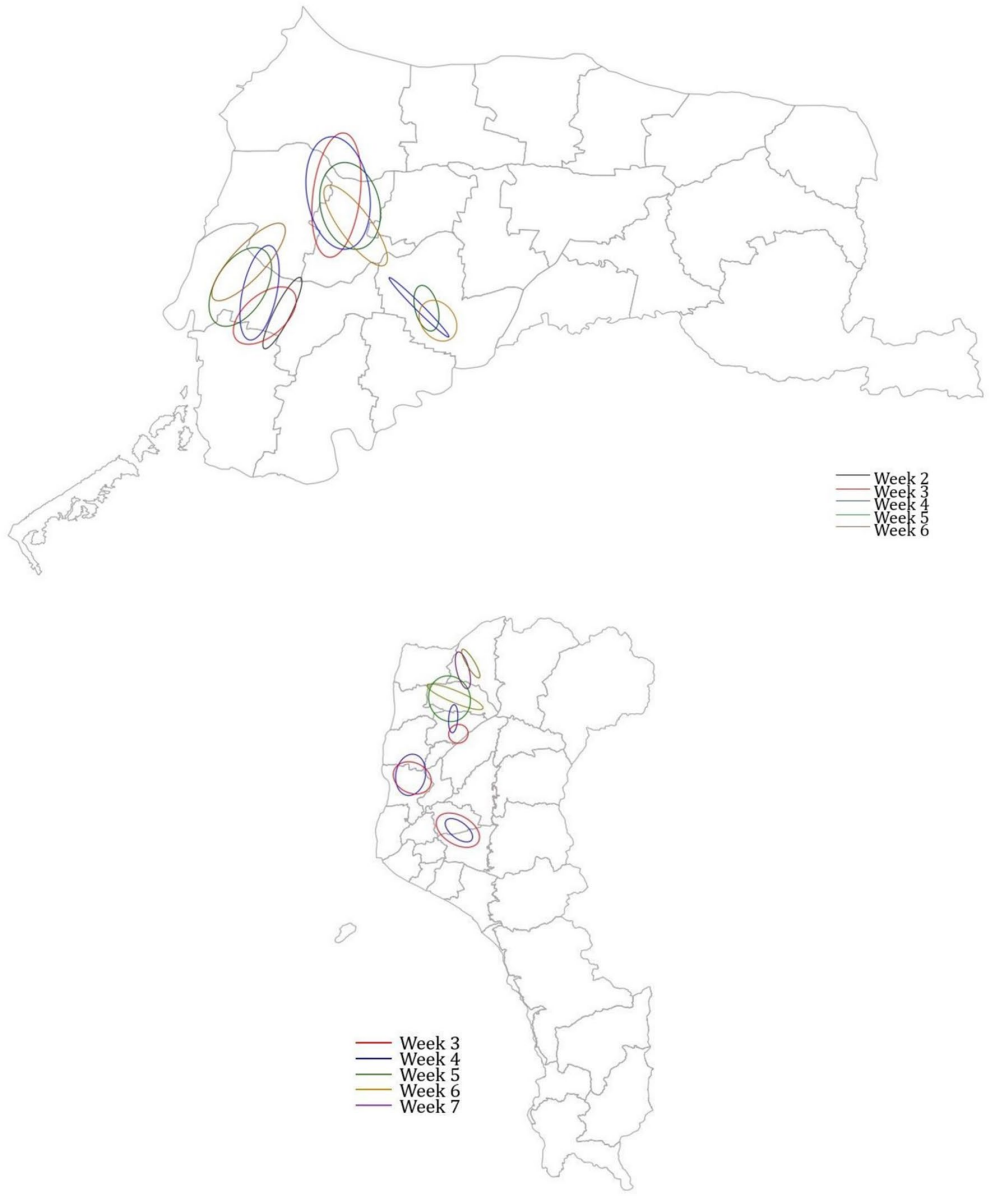
The week-by-week SDEs for individual clusters identified in YL (3 clusters, upper panel) and PT (4 clusters, lower panel) county.

Since the counties YL and PT had the most cases in January and February 2015 (Table in Fig. 2), the SDE method was used to map the dynamic change of poultry farm outbreaks within these two months on a weekly basis. According to the partitions determined by Knox statistics, the weekly SDE estimates are shown in Fig. 4. Connecting the centers of the ellipses can show possible directions of diffusion. For two counties with the most clusters identified, cluster #3 and cluster #5 in YL county had ellipse centers moving to the southeast; on the contrary, the center of cluster #4 moved towards the northwest (Fig. 4 upper panel). Although the clusters in PT county were quickly under control, cluster #9 had its center moving northward (Fig. 4 lower panel).

**Figure 5:**
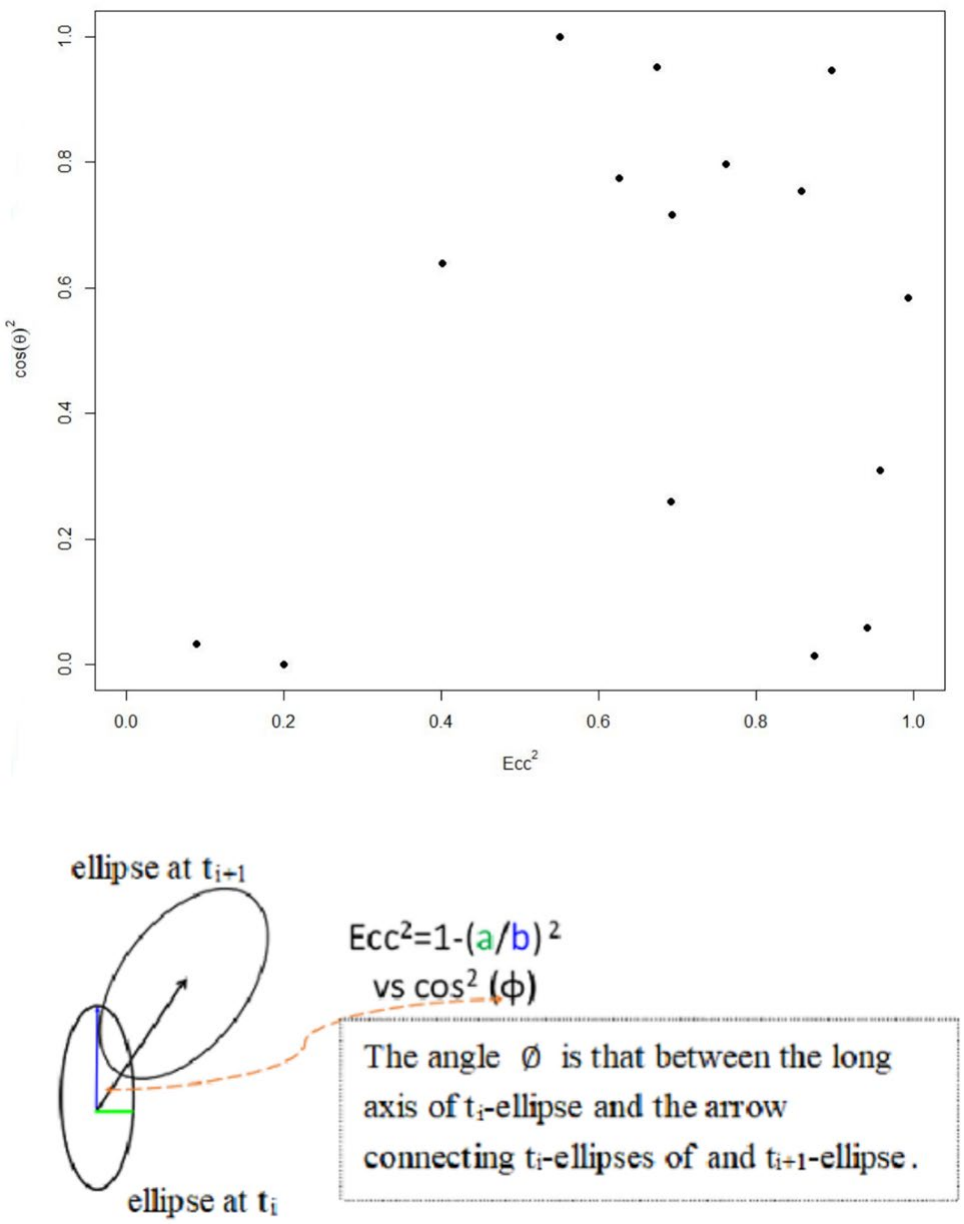
(Upper panel) The relationship between the eccentricity of the SDE in the two counties (YL and PT) and the angle ($\text{cos}(\varnothing ))$ between two SDEs depicted for 2 weeks (t_i_ and t_i+1_). Squaring makes the data points look better dispersed. Two dotted lines are taken for eccentricity = 0.8 (Ecc^2^ = 0.64) and $\text{cos}(\varnothing )$=0.8 (${\text{cos}}^{2}\left(\varnothing \right)=0.64$). (Lower panel) Illustrating the calculation of $\text{cos}(\varnothing )$.

We further examined the week-to-week outbreak diffusion by connecting the two SDE centers from two adjacent weeks (say, t_i_ and t_i+1_, i = 1,2,3…) and calculated the angle ($\varnothing$) for several t_i_-ellipses for the two counties YL and PT (lower panel of Fig. 5). Since the long axis of the ellipse may indicate a direction of subsequent viral spreading, the plot of cos^2^$(\varnothing)$ versus the square of eccentricity (Ecc^2^) implies the sequential transmission of HPAI infection events. Provided that we recognize the upper right corner of Fig. 5 (upper panel), the change in direction from this week (t_i_) to the next week (t_i+1_) means that it is positively correlated with the high eccentricity of week t_i_, and thus, defining the first quadrant as cos($\varnothing$) > 0.8 and Ecc > 0.8. 33% (= 5/15) of the week-to-week diffusion meets this expectation.

### Likelihood ratio-based spatio-temporal clustering

**Figure 6:**
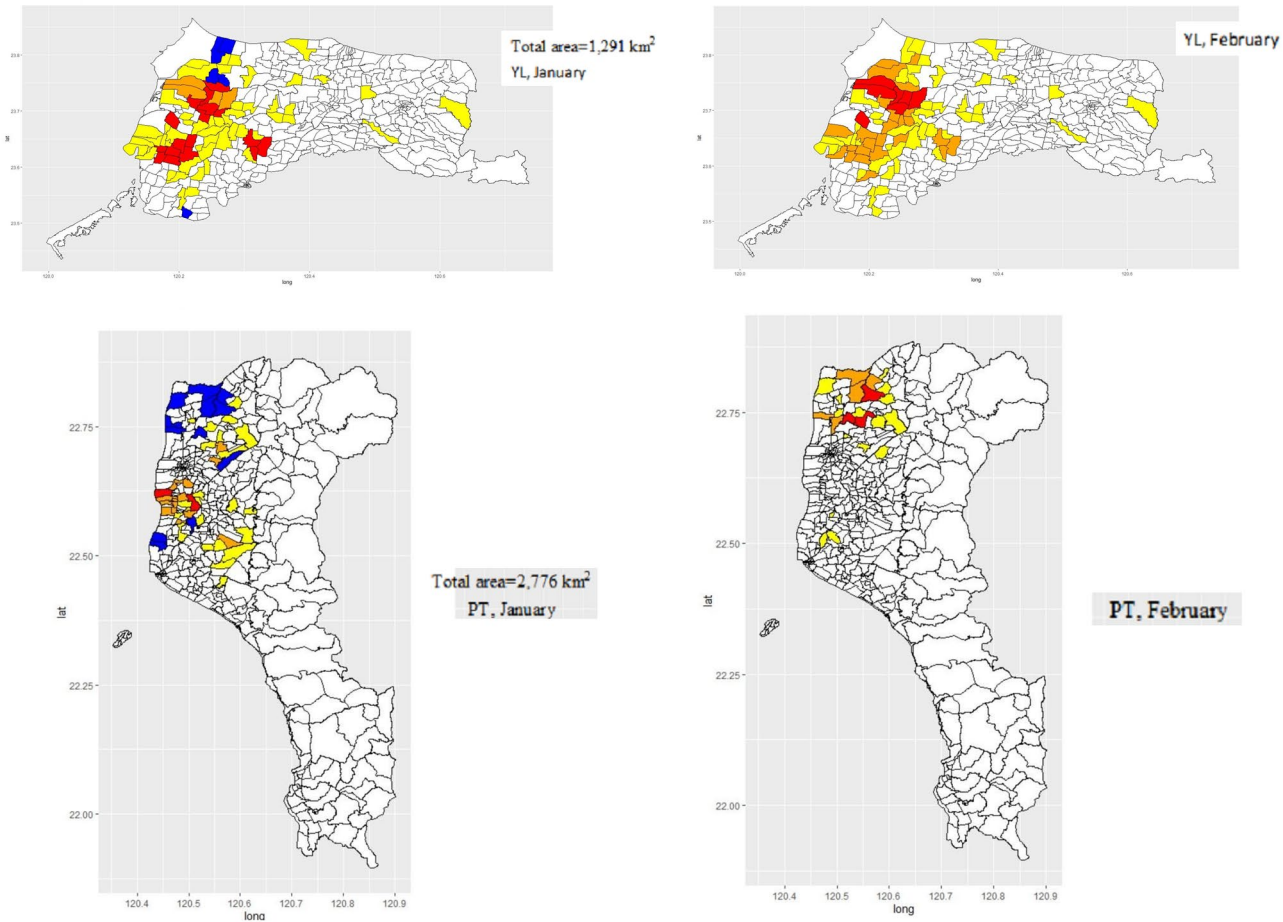
AGC maps, January and February 2015, for YL and PT counties. Resolution is set at “village-level”. Incidence levels based on AGC values are represented by different colors: white, blue, yellow, orange, and red, which mean no case (AGC value = 0), slight outbreak (AGC value < 0), moderate (0 < AGC value ≦ 2), severe (2 < AGC value ≦ 4) and extremely severe (AGC value > 4) outbreaks, respectively.

We further examined likelihood ratio-based scan statistics at the small administrative units (very close to the “village”) resolution using two different reference populations and developed a second-order aggregation index, denoted as “AGC” map, for visualizing the dynamic change of HPAI poultry farm outbreaks. Monthly AGC maps for both YL and PT counties with the highest poultry farm outbreaks were depicted for January and February 2015 (Fig. 6). Comparing the dynamic changes in the aggregation values of the AGC index within two months also reveals the direction of transmission within each “independent” cluster.

## Discussion

### Knox-based spatial mapping

Spatial scan statistics is a widely used approach to detect spatial clustering, although several conventional cluster analysis methods such as gap-statistics or K-means were developed^18–20^. However, the use of spatial scan statistics requires model calibration by appropriately tuning the time–space window^11,17,21^. Knox (1964) proposed a method that can test the temporal and spatial interaction of infectious disease events without using any pre-defined value of distance or time to determine the clusters^10,11^. Knox statistics can be calculated by pairing all possible data points in a clearly defined geographic area and time interval. The pairs that are “close” in space and time can be tested^10^. Also, Knox statistic doesn’t require the prior knowledge of reference population in spatial scan statistics, although the problem of “population shift” may exist^16,22^. Due to the incompleteness of the data, in our research, this problem was ignored by assuming the population of poultry farms remains stable within the reference population (i.e., Taiwan or smaller administrative regions such as counties or villages), despite the routine culling of the infected premises (IP) during the outbreaks. Adjustment for population-shift bias will be pursued in the future study^10,11,23^.

Another limitation of using Knox-based algorithm is that no rigorous statistical method has been developed as a formal test to give an exact or approximate (in terms of large sample theory) p-value to assess the significance of space–time two-dimensional searching for the optimal cutoff points, although Mantel (1967) proposed to use the permutation methods^16^. The optimality in Knox-based clustering refers to a maximal association measure (here, odds ratio) between the two factors space and time. For the case of one-dimensional search for optimal selection, statistical literature called it a problem of “maximally selected chi-square statistic”^24^. Their derivation led to the “sup” (supremum) of a *Brownian* bridge process (or a tied-down *Brownian* motion process) as a testing statistic, which has a well-known asymptotic distribution and a prepared table for the significance level (p-value). However, Miller and Siegmund’s result cannot be directly used in our study, because their search for maximizing the association is one-dimensional, whereas our searching for optimal association is “two-dimensional” (space plus time). Turnbull et al. (1990) has also made a similar attempt to find unrelated clusters of chronic diseases such as leukemia, but the method is computationally demanding^25,26^.

Developing a statistical test is not our purpose here as we only aim to identify a reasonable clustering pattern exhibition. Based on the setting of Knox’s, Barton and David (1966) proposed an “intersection” approach to obtain spatiotemporal clustering^27^. They proposed to connect event pair line segments with temporal and spatial clustering to form a “temporal map” and “spatial map”, respectively. Finally, these maps are combined to get a spatiotemporal clustering graph^16^. This approach was reasonable but not always easy to achieve because in the region where the number of outbreaks is large, and this is our case, thousands of lines are entangled, and visually discriminating different clusters could be difficult. We showed in this study that the Knox-based approach could still display spatiotemporal clusters, particularly when the outbreaks occur in multiple places (Fig. 3). When circling the major spatial clusters, each circle has a “diameter” within 3 km that is the size of the control zone established once the HPAI-infected farm is identified in Taiwan. When an IP is reported, all poultry from that particular IP will be culled, and all farms within the 3 km radius of that IP will be targeted for intensive surveillance. Therefore, additional clusters outside the 3 km control zone stand for the spreading of HPAI viruses requiring epidemiological investigation.

### AGC mapping

Other than the Knox-based spatial clustering approach, we further developed the AGC map based on the likelihood ratio to describe spatial clustering in this study. The basic space–time statistics widely apply scan statistics with Poisson/binomial distribution to compare the disease risk within and outside the scanning window. Instead of arbitrary specification, a trial-and-error approach to explore spatial and temporal parameters, including the maximum spatial window (25 and 50% of the population at risk), the maximum temporal window and *p* values (< 0.05 and < 0.001), is necessary. In contrast with the traditional approach of searching spatial and temporal space for clustering, the likelihood ratio statistics constructed in this study considers two “reference populations” to serve as the basis for statistical testing on global and local spatial clustering. Then, the λ-values from likelihood ratio scan statistics or AGC index are used to get the second-order clustering for visualization of spatio-temporal clustering changes. Such inherent property of AGC index has been largely ignored and was demonstrated as a visual tool for spatio-temporal mapping here. Compared to the basic space–time statistics, which deliver the outputs of the first most likely cluster and secondary clusters (if *p* value < 0.05 with no geographical overlap), we propose a map based on drawing the AGC index, which can capture the aggregation pattern of disease clusters, and therefore is very useful for displaying hotspots. That is, the aggregation of those sub-regions with higher ${R}_{j}$ or AGC index is called hotspots. The identified major clusters are similar in both Knox-based and AGC mapping methods (Figs. 3, 6). Although the AGC map inevitably depends on the choice of the critical value of the AGC index, the difference between the two results is small. These major spatial clusters or hotspots could share common environmental risk factors contributing to the poultry farm outbreaks by HPAI, which we published previously^2^. By monthly depicting AGC maps, the changes in the hotspot pattern over time also provide clues on the direction of HPAI viral transmission. Tildesley et al. provided an extensive discussion on the spread of the disease and its impact^28^. If the AGC maps of different months remain unchanged, it means that the locations of hotspots are very “stable”, which imply the effectiveness of control measures implemented by BAPHIQ within the established 3-km control zone^2^. On the contrary, the AGC maps will help to identify the potential local regions, which require more stringent control measures in the future. Note that the formation of AGC maps depend on the choice of cutoff point for the number of clusters. The traditional elbow method based on minimizing the overall within-cluster variation can be applied, or the more modern gap statistics can be used in the future^18,19^.

The limitation of using AGC mapping in this study is the difficulty to describe the statistical property of the AGC index since it is far beyond the scope of the current research. In ${R}_{j}={\lambda }_{j,G}-{\lambda }_{j,g}$, each  is a “likelihood ratio”, and the statistical (large sample) properties are well known for the likelihood ratio statistics. However, the calculation of the variance of ${R}_{j}$ (AGC index) inevitably involves the correlation between ${\lambda }_{j,G} \text{and} {\lambda }_{j,g}$, which is not an easy task although it can be pursued by a bootstrap re-sampling scheme^29^. This will not be presented in this paper.

### Visual tools for mapping viral transmission direction

Our initial attempt was to apply the regression model proposed by Zinszer et al. to estimate the local spreading of the Ebola epidemic^30^. The model points out that for an outbreak that occurs at calendar time T_i_ and location (${X}_{i}$, ${Y}_{i}$), there is a corresponding explanatory variable (can be a vector) Z_i_, T_i+1_ is the time of the next (Ebola) outbreak, so the “interval time” τ_i_ = T_i+1_ − T_i_ between two adjacent events can be modeled as:

4 $${\tau }_{i}={\beta }_{0}+{\beta }_{1}{X}_{i}+{\beta }_{2}{Y}_{i}+{f}_{1}\left({X}_{i}\right)+{f}_{2}\left({Y}_{i}\right)+{\gamma {^{\prime}}Z}_{i}+{\varepsilon }_{i},$$

where ${f}_{1}$ and ${f}_{2}$ are two functions that could be chosen as polynomials. The parameters ${\beta }_{1}$ and ${\beta }_{2}$ are interpreted as the reciprocal of the transmission rate in the X and Y directions respectively and are usually used as the longitude (X) and latitude (Y) of the outbreak event indexed by “i”. The magnitude of changes in X and Y is random, implying the velocity (speed plus direction) of transmission. If the time interval is fixed at 1 week, the change of velocity by week visualizes the change in direction and speed of viral transmission by considering the first two events that occurred in each township to estimate the direction according to the formula. However, we found that Zinszer’s approach may not be suitable here (Supplementary Fig. S1) for the following reasons. First, multiple events may occur in a short period, and the location of the earliest event may sometimes be difficult to determine due to delay of case reporting, recall bias, or heterogeneous and mild outbreak symptoms. Second, when the transmission space in question is relatively small, the variability of velocity (i.e. transmission speed) will increase. Thirdly, it is also difficult to distinguish transmission direction within and between spatial clusters, where different local and non-local factors have different impacts on the outbreak events.

Although there is no universally feasible method to estimate the direction of transmission, the use of SDE to visualize the geographical distribution of a series of social, biological, or environmental events remains attractive^31–34^. In this study, SDE is applied to individual spatial clusters, defined by the Knox method, to reveal its local transmission by week. By connecting the consecutive centers of weekly SDEs, the direction of transmission can be easily visualized, which may imply the playing roles of local factors, such as wild bird movement, transport vehicles, human activities, or other meteorological factors acting within the spatial clusters^7,35–39^. Other non-local factors, such as factors related to poultry market supply networks or the long-distance movement of specific bird species, contributing to the HPAI transmission between spatial clusters can be investigated and differentiated from the local factors^40–42^. Careful identification of influencing factors can help precautionary measures, public health control, and prevent further outbreaks.

In conclusion, a Knox-based combined SDE visualization tool was developed in this study to identify the spatio-temporal clustering of poultry farm HPAI outbreaks in Taiwan. Such a method is anticipated to be applicable to other infectious diseases and countries. On the other hand, AGC maps in regular intervals provide a quantitative risk at the regional level, and its dynamic change further indicates the direction of transmission. Compared to the one-stage aggregation approach, Knox-based and AGC mapping two-stage algorithms were more sensitive in small-scale spatio-temporal clustering. Our previous study suggested high poultry farm density, poultry heterogeneity index, non-registered waterfowl flock density and high percentage of cropland coverage are strongly associated with the spatial clustering of H5N2 and H5N8 circulations during 2015 and 2017 among poultry farms in Taiwan^2^. The direction of dynamic viral transmission among poultry farms identified in this study could further indicate the local environmental factors, such as highway systems for vehicle transportation and habitats overlapping with wild birds, which require further investigation in the future.

## Supplementary Information

Supplementary Information 1.

Supplementary Information 2.
