# Supplementary material for: Two-stage algorithms for visually exploring spatio-temporal clustering of avian influenza virus outbreaks in poultry farms
Source: Sci Rep. 2021 Nov 19;11:22553. doi: 10.1038/s41598-021-01207-4 (PMC8604947; doi:10.1038/s41598-021-01207-4)

**Supplementary Figure S1**: Township-wise estimates of local spreading direction using Zinszer model. Upper panel: Yun-Lin County. Lower panel: Ping-Tung County. The overall direction of a township is estimate by an angle; a larger angle represents a faster local spreading.


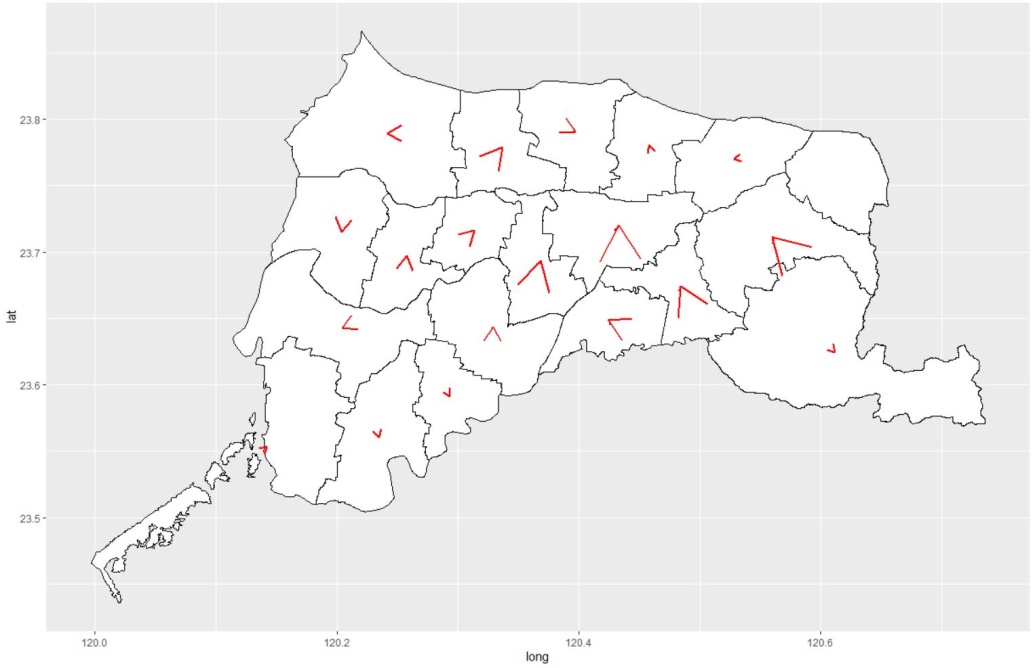


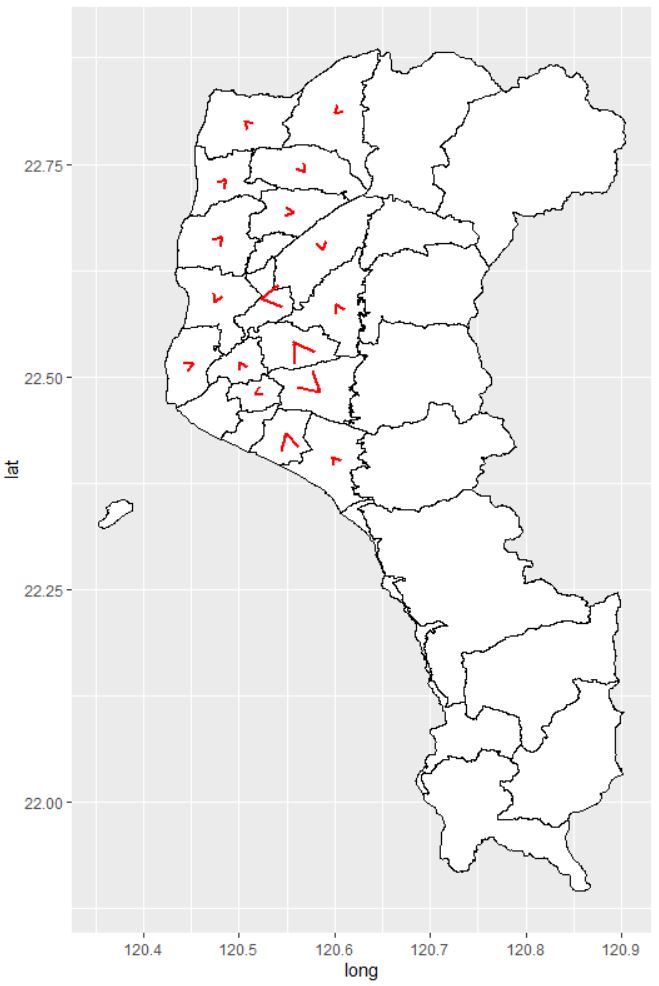

Supplement: Supplementary file 1 — Supplementary Information 1. [file 41598_2021_1207_MOESM1_ESM.docx]
