# Supplementary material for: Two-stage algorithms for visually exploring spatio-temporal clustering of avian influenza virus outbreaks in poultry farms
Source: Sci Rep. 2021 Nov 19;11:22553. doi: 10.1038/s41598-021-01207-4 (PMC8604947; doi:10.1038/s41598-021-01207-4)
Supplement: Supplementary file 2 — Supplementary Information 2. [file 41598_2021_1207_MOESM2_ESM.docx]

Two-stage algorithms for visually exploring spatio-temporal clustering of Avian influenza virus outbreaks in poultry farms

Hong-Dar Isaac Wu^1^and Day-Yu Chao^2,*^

1. Department of Applied Mathematics and Institute of Statistics

2.Graduate Institute of Microbiology and Public Health,

National Chung Hsing University, Taiwan.

Determining the clusters, major or minor, depends on the process of choosing cutoff points in space (distance; “d” in km) as well as in time (“t” in day). Let us show briefly the “process” in Yun-Lin County by exhibiting three time-cutoffs (7, 10, and 40 days) and three distance-couoffs (1, 2, and 3 kilometers). The pattern of clusters changes as follows:

| **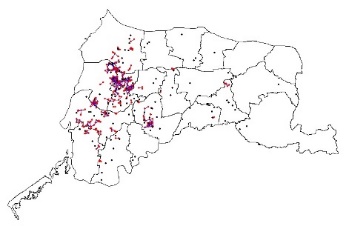d=1 and t=7** | **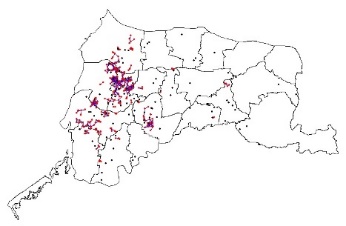d=1 and t=10** | **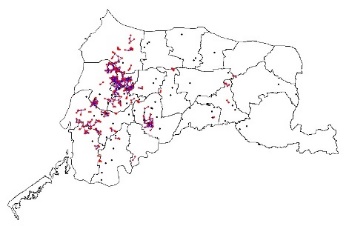d=1 and t=40** |
| --- | --- | --- |
| **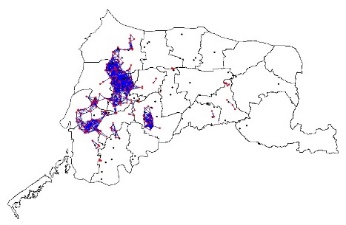d=2 and t=7** | **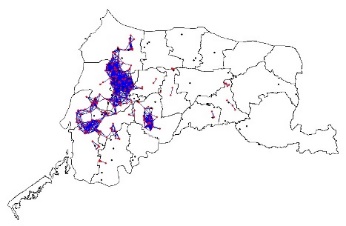d=2 and t=10** | **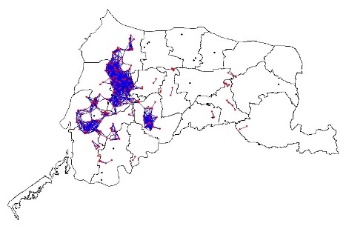d=2 and t=40** |
| **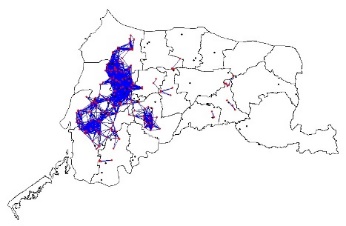d=3 and t=7** | **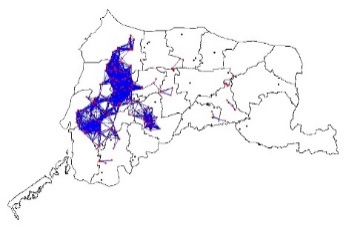d=3 and t=10** | **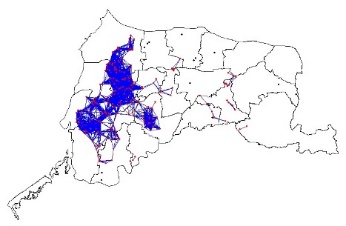d=3 and t=40** |

The patterns of Ping-Tung are similar. One can conclude from these clustering patterns that: smaller “d” and “t” make the dots (outbreaks) and line-segments more disperse; and larger “t” and “d” unavoidably recognize as much points as possible to treat them as the same cluster(s). The process of plotting these clustering patterns suggested that, for Yun-Lin, **3 major clusters** may be appropriate. If we rely on the odds ratio estimate, Yun-Lin gives the maximum odds ratio (1.81) for d=2 and t=7; and Ping-Tung gives the maximum odds ratio (4.00) for d=3 and t=9. [As we can see in the above figures, “d=1” usually cannot produce tangled segments to form visually clear “bulks”. We confine our search for d>=2.] Generally, in our study, the configurations between d={2,3} and t={7,8,9} are all possible candidates. An overview for the entire 6-county map makes us choose the cutoff (d,t)=(3,7), as was displayed in our Figure 3.
